# Dopaminergic Modulation of Synaptic Plasticity, Its Role in Neuropsychiatric Disorders, and Its Computational Modeling

**DOI:** 10.32598/bcn.9.10.125

**Published:** 2019-01-01

**Authors:** Mojtaba Madadi Asl, Abdol-Hossein Vahabie, Alireza Valizadeh

**Affiliations:** 1. Department of Physics, Institute for Advanced Studies in Basic Sciences (IASBS), Zanjan, Iran.; 2. School of Cognitive Sciences, Institute for Research in Fundamental Sciences (IPM), Tehran, Iran.

**Keywords:** Dopamine, Neuromodulation, Neuropsychiatric disorders, Synaptic plasticity

## Abstract

Neuromodulators modify intrinsic characteristics of the nervous system in order to reconfigure the functional properties of neural circuits. This reconfiguration is crucial for the flexibility of the nervous system to respond on an input-modulated basis. Such a functional rearrangement is realized by modification of intrinsic properties of the neural circuits including synaptic interactions. Dopamine is an important neuromodulator involved in motivation and stimulus-reward learning process, and adjusts synaptic dynamics in multiple time scales through different pathways. The modification of synaptic plasticity by dopamine underlies the change in synaptic transmission and integration mechanisms, which affects intrinsic properties of the neural system including membrane excitability, probability of neurotransmitters release, receptors’ response to neurotransmitters, protein trafficking, and gene transcription. Dopamine also plays a central role in behavioral control, whereas its malfunction can cause cognitive disorders. Impaired dopamine signaling is implicated in several neuropsychiatric disorders such as Parkinson’s disease, drug addiction, schizophrenia, attention-deficit/hyperactivity disorder, obsessive-compulsive disorder and Tourette’s syndrome. Therefore, dopamine plays a crucial role in the nervous system, where its proper modulation of neural circuits may enhance plasticity-related procedures, but disturbances in dopamine signaling might be involved in numerous neuropsychiatric disorders. In recent years, several computational models are proposed to formulate the involvement of dopamine in synaptic plasticity or neuropsychiatric disorders and address their connection based on the experimental findings.

## Highlights

- Dopamine signaling is one of the most important factors that affects the synaptic plasticity in the nervous system.
- Impaired dopamine signaling is thought to be involved in several neuropsychiatric disorders.
- Computational models incorporate dopamine as an additional factor to account for reward-related learning.

## Plain Language Summary

Plastic neuronal networks in the nervous system are highly adaptive. In such networks, neuronal activity patterns shape and reshape the emerging connectivity patterns between interconnected neurons. The classical view of synaptic plasticity is mainly based on the stimulus-related learning that depends on the firing activity of pre- and post-synaptic neurons. However, recent experiments have revealed the crucial role of dopamine signaling in the reward-related learning. While proper signaling of dopamine has a wide variety of important effects on the function of the nervous system mediated by synaptic plasticity, interferences in its signaling are involved in several neuropsychiatric disorders. Here, we review theoretical and computational aspects of dopamine signaling in synaptic plasticity and its possible involvement in several brain diseases.

## 1. Introduction

The functional properties of neurons can be tuned based on the received input. The flexibility of the nervous system to adjust its function based on the input might even affect the structural connectivity patterns of the system, which is conceivable by the effect of synaptic plasticity on the global structures of neuronal networks (Bayati & Valizadeh, 2012; Bayati, Valizadeh, Abbassian, & Cheng, 2015; Madadi Asl, Valizadeh, & Tass, 2017). However, neuromodulators modify synaptic transmission and integration mechanisms, which in turn regulate intrinsic properties of the neural circuits including excitability of the pre- and postsynaptic neurons, probability of Neurotransmitters (NTs) release, and receptors’ response to neurotransmitters on multiple time scales (Marder & Thirumalai, 2002; Tritsch & Sabatini, 2012; Nadim & Bucher, 2014).

Neuromodulation is referred to the modulation of the intrinsic properties of nervous system that might affect the performance of cells (Kaczmarek & Levitan, 1987; Krames, Peckham, & Rezai, 2009; Dayan, 2012; Marder, 2012). Therefore, neuromodulation controls the functional activity of the nervous system. The underlying mechanism involves targeted release of neuromodulators such as Acetylcholine (ACh), Dopamine (DA), Norepinephrine (NE), and serotonin (5-HT), which can attach to receptors of the postsynaptic neuron. The synaptic modulation of neuromodulators can be performed in multiple time scales, which affects both short-term and long-term dynamics of the nervous system. Among these neuromodulators associated with synaptic plasticity mechanisms, DA is the most important one involved in behavior and learning process (Montague, Hyman, & Cohen, 2004).

The role of DA in the Central Nervous System (CNS) is approved by numerous studies (Carlsson, Lindqvist, Magnusson, & Waldeck, 1958; Carlsson, 1959; Greengard, 2001), which is associated with attention, learning, and motivation. DA is involved in Reinforcement Learning (RL) that plays a significant role in the regulation of cognitive functions including working memory and decision making (Montague, Dayan, & Sejnowski, 1996; Berridge & Robinson, 1998; Dayan & Balleine, 2002; Tsai et al., 2009; Shohamy & Adcock, 2010; Flagel et al., 2011; Collins & Frank, 2016; Schultz, 2016).

In brain networks, the synaptic strengths can be modified based on the activity of neuronal populations that induce Long-Term Potentiation (LTP) and Long-Term Depression (LTD), which can also be affected by the action of several modulators such as DA. In dorsal striatum, DA signaling through specific pathways is required both for LTP and LTD (Pedrosa & Clopath, 2017).Experimental studies indicate that DA is involved in synaptic plasticity and memory mechanisms (Jay et al., 2004). It is shown that LTP of hippocampal-prefrontal synapses is driven by the level of mesocortical dopaminergic activity (Jay et al., 2004).

While the appropriate function of DA signaling through nervous system leads to flawless synaptic plasticity and cognitive functions, malfunction of DA signaling can be potentially disadvantageous. DA’s dysfunction is engaged in several neuropsychiatric disorders such as Parkinson’s Disease (PD), drug addiction, schizophrenia, Attention-Deficit/Hyperactivity Disorder (ADHD), Obsessive-Compulsive Disorder (OCD), and Tourette’s Syndrome (TS) (Frank, 2005; Frank, Santamaria, O’Reilly, & Willcutt, 2007; Volkow, Fowler, Wang, Swanson, & Telang, 2007; Guillin, Abi-Dargham, & Laruelle, 2007; Dagher & Robbins, 2009; Maia & Frank, 2011; Montague et al, 2012; Lee, 2013; Wang & Krystal, 2014). Patients with PD may show decreased levels of DA that could be due to the loss of dopaminergic neurons in the Substantia Nigra (SN).

Several studies on patients with PD reveal that DA is involved in motivational processes (Frank, 2005; Maia & Frank, 2011). On the contrary, patients with schizophrenia may show high DA levels that can be partially responsible for their condition. On the other hand, medical treatment of schizophrenia is often performed by the inhibition of DA’s activity (Halbach & Dermietzel, 2006). It is observed that addictive drugs enhance DA level in the forebrain structures (Di Chiara & Imperato, 1988; Volkow, Fowler, Wang, & Swanson, 2004). DA regulation mechanisms in people with ADHD are thought to be impaired (Biederman & Faraone, 2005). There are also several abnormalities in the DA signaling of some individuals with TS. Some people that are subjected to TS experience signs of OCD, while a significant percentage show symptoms of ADHD (Kandel, Schwartz, & Jessell, 2000).

**Figure 1.**
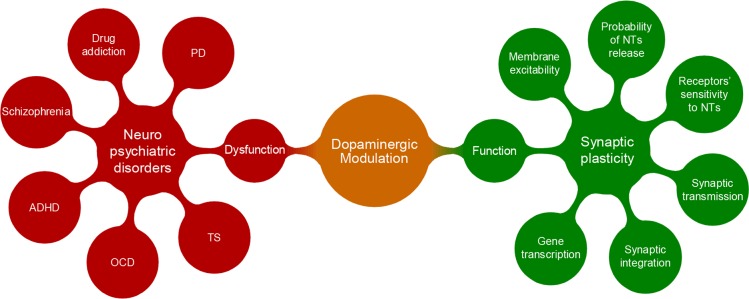
Neuromodulation by DA The most significant consequence of proper dopaminergic modulation is the modification of synaptic dynamics associated with the regulation of synaptic and intrinsic properties of the neuron. In contrast, interruption in dopaminergic system is thought to be involved in several neuropsychiatric disorders.

Figure 1 classifies some features of neuronal dynamics that can be subjected to dopaminergic modulation of synaptic plasticity, and on the contrary, several neuropsychiatric disorders associated with disturbances in DA’s function. The current study briefly reviewed the effect of DA signaling on the cellular-level mechanisms of synaptic plasticity including the change in intrinsic properties of cells and introduced neuropsychiatric disorders which are thought to be triggered by malfunctioning of DA circulation in the nervous system. However, the current review study aimed at summarizing various computational models proposed in order to formulate the involvement of DA in the synaptic plasticity machinery, as well as its possible computational roles in several neuropsychiatric disorders.

## 2. Cellular-Level Dopaminergic Modulation of Synaptic Plasticity

The substantial source of DA generation in the mammalian CNS is characterized by dopaminergic neurons reside in the midbrain (Chinta & Andersen, 2005).The Substantia Nigra pars compacta (SNc) and Ventral Teg-mental Area (VTA) are two important centers that provide the significant amount of DA to the Basal Ganglia (BG) and forebrain embeds most of DA neurons in the CNS. In spite of their minority, midbrain dopaminergic neurons may have significant impact on the large-scale behavioral functions of the brain (Tritsch & Sabatini, 2012). The signaling of DA over nervous system is facilitated via a family of metabotropic G-Protein-Coupled Receptors (GPCRs) referred to as D1-D5 receptors (Cools & Van Rossum, 1976).

Typically, these DA receptors are grouped into two subsets based on their intrinsic characteristics including D1-like and D2-like types. D1-like type of DA receptors consists of D1 and D5 receptors, while D2-like family comprises D2-D4 receptors. One of the most important DA pathway signaling is by BG circuitry that is crucial for stimulus-response learning (Cohen & Frank, 2009). BG is involved in action selection (Maia & Frank, 2011) and its impairment is thought to be involved in several neuropsychiatric conditions that are linked to behavioral control (Stocco, Lebiere, & Anderson, 2010). The BG circuitry consists of direct (Go), indirect (NoGo), and hyperdirect (global NoGo) pathways from cortex to BG outputs (Albin, Young, & Penney, 1989; DeLong, 1990).

Neurons in direct pathway are influenced mostly by D1-like receptors, which boost desired actions in the ongoing state, while NoGo neurons are subjected to D2-like receptors and restrain actions that are inappropriate for the state (Maia & Frank, 2011). Therefore, the neuromodulatory signaling can regulate the intrinsic properties of cells at molecular level including the modulation of excitability of the pre- and postsynaptic neurons, probability of neurotransmitters release, and receptors’ response to neurotransmitters, which in turn determines the responsiveness of synaptic dynamics (Harris-Warrick & Marder, 1991; Marder, 1998; Jay, 2003).

Neurons can modify their intrinsic membrane characteristics based on the received input that can adjust the membrane excitability and its responses to synaptic input, which is under modulatory signals (Hille, 2001). Neuromodulators such as DA can modify the excitability of neurons by regulating the voltage- or ligand-gated channels (Kaczmarek & Levitan, 1987; Harris-Warrick & Marder). For instance, it is observed that DA exerts a significant influence on Striatal Projection Neurons (SPNs) intrinsic excitability (Nicola, Surmeier, & Malenka, 2000). The modulation of intrinsic activity properties of neurons may have significant impact on the behavioral state. In mammalian thalamus, the modulatory signaling leads to a transition between different patterns of firing frequency that might be related to the transition between awake and sleep phases (McCormick & Pape, 1990; McCormick, 1992; Steriade, McCormick, & Sejnowski, 1993).

DA signaling is also involved in the release process of neurotransmitters, which can change the release probability of both pre- and postsynaptic neurons by modifying their receptor signaling (Tritsch & Sabatini, 2012). However, the effects of DA on the transmitter release can be complicated since they highly depend on the context in which they are working. On the other hand, the major aim of DA signaling is to modify the postsynaptic neurotransmitters.

DA along with other neuromodulatory signals regulates function and trafficking of GABA, NMDA, and AMPA receptors through different pathways (Tritsch & Sabatini, 2012). Several physiological and computational studies revealed the ability of DA signaling to modify the ionic and synaptic currents (Sawaguchi, Matsumura, & Kubota, 1990a; Sawaguchi, Matsumura, & Kubota, 1990b; **Yang & Seamans, 1996;** Durstewitz, Seamans, & Sejnowski, 2000). DA’s activity may enhance the GABAergic transmitter release and related currents in the Prefrontal Cortex (PFC) **(Zhou & Hablitz, 1999)**. On the other hand, DA signaling may reduce the Excitatory Postsynaptic Potential (EPSP) amplitude associated with AMPA- and NMDA-like synaptic currents (Cepeda, Radisavljevic, Peacock, Levine, & Buchwald, 1992; Kita, Oda, & Murase, 1999).

Although the detailed molecular-level mechanism governing the DA signaling in brain is unknown, several studies indicated that DA is able to regulate the physiological properties of different ionic and synaptic currents of PFC neuronal networks related to working memory tasks (Law-Tho, Hirsch, & Crepel, 1994; Yang & Seamans, 1996; Gulledge & Jaffe, 1998) or behavioral performance during RL in the PFC (Sawaguchi et al., 1990a; Sawaguchi et al., 1990b; Williams & Goldman-Rakic, 1995). It is demonstrated that certain DA levels in PFC are involved in the optimal control of visual cortical signals (Noudoost& Moore, 2011). There is also strong supporting evidence implicating that DA-related regulation of synaptic plasticity and neural signaling exist in several cortical layers (Soltani, Noudoost, & Moore, 2013).

## 3. Computational models of DA-modulated synaptic plasticity

Synaptic plasticity is a process that adjusts synaptic strengths according to the correlated activity of preand postsynaptic firings. Based on Hebb’s rule (Hebb, 1949), simultaneous activation of pre- and postsynaptic neurons leads to an increase in the synaptic strength of the synapse connecting them. Spike-Timing-Dependent Plasticity (STDP) (Gerstner, Kempter, van Hemmen, & Wagner, 1996; Markram, Lübke, Frotscher, & Sakmann, 1997; Bi & Poo, 1998; Song, Miller, & Abbott, 2000) modifies synaptic strengths according to pre- and post-synaptic spike pairs related to Hebbian learning rules.

STDP rule works based on the stimulus-stimulus learning protocol where naturally ignores the possible intervention of neuromodulatory signals that can be due to the events such as reward or punishment (Gerstner, Kistler, Naud, & Paninski, 2014; Frémaux & Gerstner, 2016). However, several studies revealed the crucial role of neuromodulators such as DA in novelty and reward-related processes (Berridge & Robinson, 1998; Dayan & Balleine, 2002; Schultz, 2002; Yu & Dayan, 2005; Tsai et al., 2009; Shohamy & Adcock, 2010; Flagel et al., 2011; Collins & Frank, 2016; Schultz, 2016). The involvement of DA signaling in synaptic plasticity and learning process can be taken into account by stimulus-reward learning protocol or the so-called neoHebbian formalism that depends on pre- and postsynaptic activities and the influence of neuromodulators (Montague et al., 1996; Lisman, Grace & Duzel, 2011; Frémaux & Gerstner, 2016).

### 3.1. DA-induced reinforcement learning

Several RL models are proposed in order to elucidate the role of DA modulation in behavioral and neural signaling during different learning tasks (Maia & Frank, 2011; Collins & Frank, 2014).These models such as Temporal-Difference RL (TDRL), or Temporal-Difference STDP (TD-STDP), assign a value to each specific action (Suttonand & Barto, 1998; Schultz, 2002; Frémaux & Gerstner, 2016). This assigned value can be altered by the induction of DA-related reward prediction errors in order to trigger learning. The choice between various actions is random that is completed by the comparison between different states. The mathematical formulation of RL rules are discussed shortly. Although, these models can justify different sets of data, each one has its own shortcomings in taking into account the modulatory influence of DA on the selected choice.

### 3.2. Mathematical formulation of DA-modulated Hebbian plasticity

The Hebbian learning rules work based on the joint activity (e.g. rate or timing) of pre- and postsynaptic neurons. However, introducing a new factor such as a neuromodulator defines a synaptic plasticity rule referred to as three-factor rule (Lisman et al., 2011; Frémaux & Gerstner, 2016) that can be formulated as follows:

(1) $$\dot{g}(t)=F(M,\;\text{pre},\text{post})$$

where ġ denotes the change in the strength of synapse connecting presynaptic neuron to the postsynaptic one, and parameter M is the modulatory agent, which can be a reward-related DA signal, for example. The function F denotes the nature of the learning rule. Assuming that the neuromodulatory signal is not involved, the conventional forms of synaptic plasticity rules can be recovered. One example is the classic STDP rule where the learning window F can be written as follows (Gerstner et al., 1996; Markram et al., 1997; Bi & Poo, 1998; Song, 2000):

(2) $$F(\Delta t)=A_{\pm }\text{sgn}(\Delta t)\text{exp}(-|\Delta t|/\tau_{\pm })$$

where A_±_ and τ_±_ are amplitude and time constant of the synaptic modification. Δt=t_post_-t_pre_ denotes the difference between the timiΔng of pre- and postsynaptic firings.

The learning window F can be determined by several learning rules such as reward-modulated STDP (RSTDP) model (Farries & Fairhall, 2007; Florian, 2007; Izhikevich, 2007), which modifies synaptic strengths in the mean over several trials developed by the modulation of standard STDP with a reward-related DA term. The average of recent joint spike-timings in a conventional Hebbian STDP can be denoted by a function H (pre, post) (Frémaux & Gerstner, 2016). The modification of synaptic strengths is performed whenever the modulator M=R(t)-b, indicates a deviation of the reward R(t) from the baseline b (expected reward):

(3) $$\dot{g}(t)=[R(t)-b]\times H(\text{pre},\text{post})$$

where the baseline can be set equal to the average reward b=(R(t)). The other types of learning protocols are spike-timing-dependent versions of TD learning, namely TD-STDP, where the change in synaptic strengths occurs after a single trial. Experimental studies show a reliable link between TD error arising in RL and activity patterns of dopaminergic neurons during reward-related experiments (Schultz, 2002; Schultz, 2016).It is shown that TD error, δ(t), also known as prediction error, can be written as follows (Montague et al., 1996):

(4) δ(t)=R(t)+V(t)−V(t−1)

where R(t) is reward, and V(t) is value expected at time t. Therefore, the explicit form of TD learning rule takes the form (Frémaux, Sprekeler, & Gerstner, 2013):

(5) $$\dot{g}(t)=\delta (t)\times H(\text{pre},\text{post})$$

where δ(t) is TD error. Therefore, the role of dopaminergic neurons can be simulated by reward function R(t) in a TD model. However, a learning process could be triggered by a surprise signal that demonstrates the observed novelty compared to expected value, which apparently does not obey reward-based learning rules. In this case, the change in synaptic strengths depends on a variable that represents surprise S(t) (Rezende & Gerstner, 2014):

(6) $$\dot{g}(t)=S(t)\times H(\text{pre},\text{post})$$

where this surprise term represents novelty with respect to the expected value and could be induced by the activity of dopaminergic neurons.

## 4. The Computational Role of DA in Neuro-psychiatric Disorders

The crucial role of dopaminergic modulation in several brain processes and its participation in a variety of brain functions including cognitive functions and behavioral control indicates that impaired DA signaling can be potentially involved in several major brain disorders. Therefore, the study of clinical features of DA signaling can be a precious tool to reveal the complicated nature of dopaminergic modulation in the brain. Dysfunction of the dopaminergic circuitry can change the normal function of the nervous system, and as a result may cause several neurological and psychiatric disorders. However, along with the clinical applications of dopaminergic modulation of the nervous system, the role of DA in neuropsychiatric disorders can be simulated by computational models. Computational models may enhance the treatment techniques of the neuropsychiatric disorders since different agonists or antagonists of DA can be implemented and tested in the computer simulations, which do not require a human subject.

### 4.1. Parkinson’s disease

Low levels of DA in the BG and in specific, striatum, are implicated in patients with PD that may have crucial impacts on the movement control or learning process (Frank, Seeberger, & O’reilly, 2004; Frank, 2005). It is believed that deterioration of dopaminergic neurons in PD may reduce the dopaminergic input to striatal areas, which can generate inappropriate tendency for NoGo pathway (Maia & Frank, 2011). Recently, it is shown that mean functional connectivity in patients with PD can be significantly lower than that of normal subjects (Hepp et al., 2017).PD symptoms are involved in cognitive and behavioral malfunctions due to the DA deficiency in motor system of the striatum. Although DA agonists can be used to treat patients with PD, standard therapy for PD is possible by desynchronization of pathologically strong synchronization of neurons performed using electrical High-Frequency (HF) Deep Brain Stimulation (DBS) techniques (Tass et al., 2012; Popovych & Tass, 2014; Tass, 2017).

Frankproposed a computational model in order to simulate PD with the hypothesis that the role of DA in PD lies in the reduction of DA dynamics that suppresses the learning process in BG through Go/NoGo pathways. The physiological constraints are implemented in a BG-based neural network model including Go/NoGo pathways that simulates the learning conditions. The simulation results show that the effect of low levels of DA on the PD model, and the simulated DA medication of PD are in accordance with the results observed in patients with PD. At the neural level, the model predicts that DA dynamics supports Hebbian learning by modulating synaptic dynamics in the indirect pathway: Suppressing NoGo neurons leads to LTD, while exciting them results in LTP, which is consistent with experimental observations.

### 4.2. Drug addiction

Drug addiction is entangled with difficulties in decision making which can be implicated by the potential effect of DA on the corticostriatal neurons. In fact, the crucial role of DA in reward-related process and RL generates a defective loop that the repeated use of addictive drugs can finally lead to compulsive and habitual behaviors (Montague et al., 2004). Most addictive drugs increase the level of DA in brain, therefore, the positive feedback interaction between DA neurons and such reinforcing drugs establishes a malfunctioning cycle, which results in an excessive increase in DA levels that may trigger persistent compulsive behaviors (Dagher & Robbins, 2009) implicated by a positive prediction error. Since these drugs are directly involved in DA-inspired RL, they provide a feedback loop that reinforces behavior leading to drug consumption and reveals the compulsive nature of drug addiction (Montague et al., 2004).

Redish proposed an RL-based computational model to simulate the behavioral states due to the increase of DA level using drug abuse. This model works on the basis of TDRL that its prediction error signal δ(t) is described by Equation (4) along with introducing a new variable y^d^ that denotes raising the discounting factor y by the time delay d at time t-1:

(7) $$\delta (t)=\gamma^{d}[R(t)+V(t)]-V(t-1)$$

In the TDRL model, learning ceases when the value function accurately estimates the reward, which produces no DA signal δ(t)=0. However, the enhancement of DA level due to the use of addictive drugs can be modeled by assuming that this mechanism induces a positive prediction error δ(t) that cannot be covered by changes in the corresponding value. In such a case, Equation (7) can be written as follows:

(8) $$\delta (t)=\text{max}\{\gamma^{d}[R(t)+V(t)]-V(t-1)+D(t),D(t)\}$$

where D(t) is a DA flow occurring on entry into a new state at time t. Equation (8) reduces to typical TDRL if D(t)=0, but falls to a minimum δ(t) of D(t) if D(t)>0 that produces a positive prediction error δ(t)>0. Therefore, the values cause DA to flow tends to infinity. In conventional TDRL, the corresponding values leading to a normal reward asymptotically tend to a finite value. However, in the presented model, the values resulting to drug delivery increase with no limitation, establishing an unfavorable cycle of the reinforcing factor to choose an action that leads to the corresponding states (Redish, 2004).

### 4.3. Schizophrenia

Schizophrenia can be characterized by different cognitive symptoms including impaired attention and cognitive control, which may involve excessive levels of DA in the striatum, but reduced DA amounts in PFC (Maia & Frank, 2011). However, while the main cause of the disorder is not precisely known and remains controversial, it is indicated that schizophrenia might be related to some abnormalities in the dopaminergic synapses of the brain (Howes et al., 2012).

Some results from medical treatments indicate that schizophrenia can be due to excess activity of dopaminergic synapses (Squire et al., 2012); however, other studies postulated that some cognitive dysfunctions observed in schizophrenia are related to suppression of DA activity in PFC (Braver, Barch, & Cohen, 1999). It is shown that some of the clinical symptoms of schizophrenia could be simulated by stability considerations in computational models of neural networks in the sense that fluctuation of DA level can change the stability of corresponding attractor networks in the PFC, which is thought to be involved in some of the cognitive symptoms of schizophrenia (Loh, Rolls, & Deco, 2007; Rolls, Loh, Deco, & Winterer, 2008).

Hoffman and McGlashan introduced a computational model called spurious attractor states that can explain some of the symptoms of schizophrenia by the reduction of connectivity patterns in an attractor network that partly detaches the attractor network. This can be related to the disconnection hypothesis of schizophrenia in which the connectivity of some parts of the brain becomes relatively detached (Friston, 2002).

Another different approach called connectionist model simulates several cognitive impairments that occur in schizophrenia (Braver et al., 1999). It suggests that the cognitive symptoms of schizophrenia might be caused by a failure of cognitive control. However, more realistic and physiological neural models allow researchers to simulate and elaborate the effects of dopaminergic modulation on the ionic and synaptic currents (Rolls et al., 2008).

### 4.4. Attention-Deficit/Hyperactivity Disorder (ADHD)

ADHD is associated with abnormal hyperactivity and impulsivity behaviors that may be related to the dysfunction of PFC and its cortical units (Squire et al., 2012). The common viewpoint of ADHD is that it results from a defect in inhibitory system, which leads to executive malfunctions. However, other studies show that impaired function of DA reward pathways in patients with ADHD might be involved in motivation dysfunctions in this disorder (Volkow et al., 2011). ADHD is strongly heritable and is linked to the genetic transformations that interfere with NE or DA signaling. Reduced levels of DA in the brain can lead to difficulties in the control of impulsive behavior in patients with ADHD. It is shown that damage in DA neurons of the VTA can be the cause of hyperactivity and substandard response to stress (Blum et al., 2008).

Treatments of ADHD may include medication, therapy, or a combination of them, which can be a long-term approach. Symptoms of ADHD can often be treated with proper medications, which are typically based on the increased levels of DA. Conventional therapies are targeted at alleviating symptoms by regulating different neurotransmitters including DA (Blum et al., 2008). Although, there are several treatment procedures, an appropriate treatment is dopaminergic and serotonergic releaser combination therapy that consists of agonists. However, the most important pitfall of this approach is the possibility of medicine abuse due to the activation of DA neurons in CNS reward circuits.

### 4.5. Tourette’s syndrome

TS is indicated with abrupt and capricious movements referred to as tics. It is suggested that these tics may result from abnormal excitability or plasticity procedures in the direct (Go) and indirect (NoGo) pathways (Maia & Frank, 2011).This can be interpreted as an unfavorable positive feedback loop that the tic itself enhances the possibility of its occurrence through learning procedures, which in turn increases the tendency to tic by activation of the related motor systems. Evidence shows several abnormalities in the levels of DA signaling in some individuals with TS (Buse, Schoenefeld, Münchau, & Roessner, 2013). Several hypotheses concerning the dysfunction of the DA signaling in TS are developed including the dopaminergic modulation of pre- and postsynaptic receptors and firing patterns of DA neurons (Buse et al., 2013).On the other hand, some of the individuals subjected to TS experience signs of OCD, and a remarkable number comprise symptoms of ADHD (Buse et al., 2013).

## 5. Conclusion

The current review study considered the role of neuromodulator DA in one of the most important features of nervous system, i.e. the ability of neurons to tune their functional properties based on the required conditions of inputs. DA along with other modulatory signals regulates the performance of the synapses in order to function on a stimulus-reward learning basis, far from the classical view of stimulus-stimulus learning. Therefore, DA signaling in the learning process can regulate the efficiency of synaptic plasticity and learning procedures by modulating the cellular mechanisms that control the activity pattern of neuron.

The impact of DA signaling on the synaptic plasticity at cellular level can be interpreted by understanding the molecular mechanisms that modify the intrinsic properties of cells. Such mechanisms are highly vulnerable to perturbations in their function, which can result in distorted circulation of DA signaling. In other words, the appropriate level of DA signaling across brain areas is crucial for proper function of the nervous system. However, the current review study summarized some important DA-induced computational models of synaptic plasticity associated with different learning rules. Finally, as it was mentioned earlier, disturbed DA signaling is associated with several important neuropsychiatric disorders. Although diverse disorders such as PD, drug addiction, schizophrenia, ADHD, and TS might seem to have little in common, they are all entangled with disturbances in DA’s function through nervous system.

Experimental observations may provide a comprehensive, and in some cases controversial insights to the crucial role of neuromodulators such as DA in the nervous system, but mathematical formulation and computational modeling can be useful to understand their signaling machinery. The current review, however, summarized the significant findings regarding the understanding of computational role of DA in the brain through synaptic plasticity and its involvement in several neuropsychiatric disorders, which may shed light on the complicated task of DA in the nervous system.
